# Synthesis and characterization of two new TiO_2_-containing benzothiazole-based imine composites for organic device applications

**DOI:** 10.3762/bjnano.9.67

**Published:** 2018-02-26

**Authors:** Anna Różycka, Agnieszka Iwan, Krzysztof Artur Bogdanowicz, Michal Filapek, Natalia Górska, Damian Pociecha, Marek Malinowski, Patryk Fryń, Agnieszka Hreniak, Jakub Rysz, Paweł Dąbczyński, Monika Marzec

**Affiliations:** 1 Institute of Physics, Jagiellonian University, prof. S. Lojasiewicza 11, 30-348 Krakow, Poland; 2 Military Institute of Engineer Technology, Obornicka 136 Str., 50-961 Wroclaw, Poland; 3 Institute of Chemistry, University of Silesia; 4 Faculty of Chemistry, Jagiellonian University, Gronostajowa 2, 30-387 Krakow, Poland; 5 University of Warsaw, Department of Chemistry, Zwirki i Wigury 101, 02-089 Warsaw, Poland; 6 Electrotechnical Institute, Division of Electrotechnology and Materials Science, M. Sklodowskiej-Curie 55/61 Street, 50-369 Wroclaw, Poland; 7 Hydrogen South Africa (HySA) Systems and Validation Centre, SAIAMC, University of the Western Cape, Robert Sobukwe Road, Bellville, Cape Town, South Africa

**Keywords:** azomethines, composites, HOMO-LUMO, imines, organic devices, TiO_2_

## Abstract

The effect of the presence of titanium dioxide in two new imines, (*E*,*E*)-(butane-1,4-diyl)bis(oxybutane-4,1-diyl) bis(4-{[(benzo[*d*][1,3]thiazol-2-yl)methylidene]amino}benzoate) (SP1) and (*E*)-*N*-[(benzo[*d*][1,3]thiazol-2-yl)methylidene]-4-dodecylaniline (SP2), on the properties and stability of imine:TiO_2_ composites for organic device applications were examined. The investigated titanium dioxide (in anatase form, obtained via the sol–gel method) exhibited a surface area of 59.5 m^2^/g according to Brunauer–Emmett–Teller theory, and its structure is a combination of both meso- and microporous. The average pore diameter calculated by the Barrett–Joyner–Halenda method was 6.2 nm and the cumulative volume of pores was 0.117 m^3^/g. The imine SP1 exhibited columnar organization (Col), while SP2 revealed a hexagonal columnar crystalline phase (Col_hk_). The imine:TiO_2_ mixtures in various weight ratio (3:0, 3:1, 3:2, 3:3) showed a lower energy gap and HOMO–LUMO energy levels compared to pure TiO_2_. This implies that TiO_2_ provides not only a larger surface area for sensitizer adsorption and good electron collection, but also causes a shift of the imine energy levels resulting from intermolecular interaction. Also the temperature of the phase transition was slightly affected with the increase of TiO_2_ concentration in imine-based composites. The changes observed in the Fourier transform middle-infrared absorption (FT-MIR) spectra confirmed the significant influence of TiO_2_ on structural properties of both investigated imines. Similar interactions of oxygen vacancies existing on the TiO_2_ surface with SP1 and SP2 were observed. The imine:TiO_2_ mixtures showed good air stability and reusability, which demonstrates its potential for organic device applications.

## Introduction

Understanding the interaction between various nanomaterials and small organic and polymeric molecules is of current interest for the design of new nanomaterials in several fields, for example, optoeletronics and biomedicine [1–6]. For organic devices such as solar cells or light emitting diodes, the air-stable organic compounds with special architecture are needed to obtain suitable values of the various important photovoltaic parameters (*V*_oc_ – open circuit voltage, *J*_sc_ – short circuit current density, FF – fill factor, PCE – power conversion efficiency, lifetime) and luminescence parameters (e.g., quantum yield, lifetime) [7–14]. In recent years, the efficiency of bulk heterojunction organic solar cells has shown great improvement, from 2.5 to 13%. Such rapid progress brings us closer to actual commercialization of this technology, however, still some technical issues require further attention.

Nanomaterials based on organic compounds are very promising for organic devices, offering low-cost production, flexibility, transparency, low light suitability and disposability. For example, thiazoles, a group of heterocycles with electron-rich sulfur atoms and electron-withdrawing nitrogen atoms, exhibit good hole transporting properties with a low ionization potential, making them widely used electron-accepting materials in organic solar cells [15–17]. Nowadays, imines (azomethines), being condensation products of diamine/amine and aldehyde/dialdehyde, are an emerging class of organic materials for organic photovoltaic applications due to their inexpensive production and short purification time [18–20]. In the last 10 years, photovoltaic devices containing imines and polyimines with various device architectures were investigated [19–33]. However, the bottleneck of these devices is still the low power conversion efficiency (PCE), which is currently not satisfactory for application. The highest reported value of PCE for perovskite solar cells based on azomethine with the triphenylamine and 3,4-ethylenedioxythiophene moieties was 11%, whereas the PCE value for 2,2’,7,7’-tetrakis-(*N,N*-di-*p*-methoxyphenylamine)-9,9’-spirobifluorene (Spiro-OMeTAD) was 11.9% [28]. The total cost of this azomethine, with known solubility and energy levels, is $10 per gram, which makes it a good substitute for expensive Spiro-OMeTAD [28]. Recently, asymmetrical azomethine with porphiryne moieties was tested in dye-sensitized solar cells and a PCE of 1.75% was achieved [24], while azomethine with triphenylamine, thiophene and thiazolo moieties showed a PCE of 2.2% [30].

Titanium dioxide, due to its suitable band position, non-toxicity, low cost, and simple synthesis, is an appropriate material for modification of various metals. Its chemical stability and biocompatibility plays an important role in various applications such as gas sensors, photocatalytic hydrogen generation, photovoltaic and photo-electrochemical cells, and environmental photocatalysis [34–43].

The lack of systematic studies of the influence of TiO_2_ on the thermal, structural and electrochemical properties of imines with benzothiazole moieties was the main reason for this work. In this study TiO_2_ nanoparticles were used to modify the behavior of new, symmetrical and asymmetrical imines in order to investigate the electrochemical, structural and thermal properties of the imine:TiO_2_ composites with respect to the time stability under various air conditions and the amount of TiO_2_ used. In addition, the interaction between the ground-state structure of TiO_2_ and imine has been studied by Fourier transform middle-infrared absorption measurements (FT-MIR) and cyclic voltammogram (CV) experiments. For the first time, the interaction of imines with TiO_2_ has been analyzed in terms of their geometry, type of imine moieties, different ratios and HOMO–LUMO energies. The experimental results confirm the formation of two types of ≥N–Ti bonds in imine:TiO_2_ composites as an effect of TiO_2_ interactions with the imine bond in thiazole and azomethine moieties.

## Results and Discussion

### Selected physical parameters of titanium dioxide

**Figure 1:**
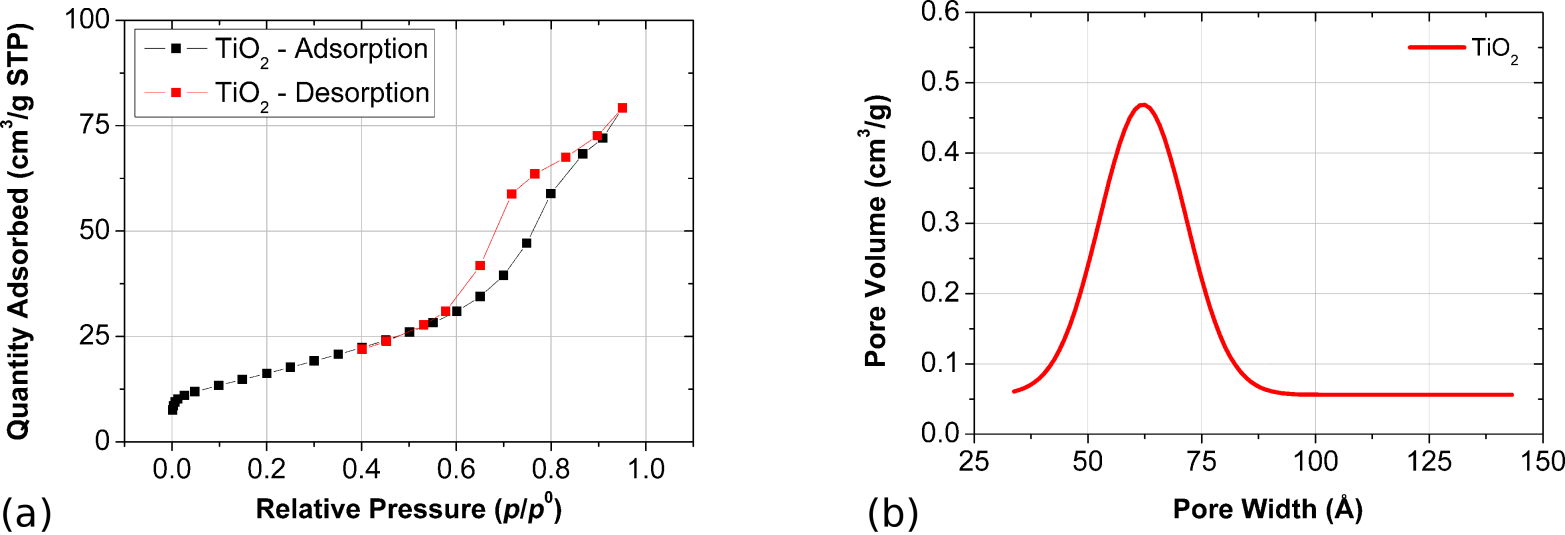
(a) Adsorption and desorption N_2_ isotherms of TiO_2_ at liquid nitrogen temperature. (b) Pore size distribution of TiO_2_ using Gaussian fitting.

TiO_2_ powder was prepared via the sol–gel technique using titanium(IV) isopropoxide (TIPO) as a precursor as was fully described in our previous paper [34]. The obtained TiO_2_ powder exhibited an anatase form, which was confirmed from X-ray diffraction (XRD) experiments [34]. The average grain size of the obtained titanium dioxide was found to be about 170 nm based on XRD, scanning electron microscopy (SEM) and atomic force microscopy (AFM) results [34]. In this paper we investigated selected physical parameters of TiO_2_ such as surface area and porosity. To calculate the surface area, the Brunauer–Emmett–Teller (BET) theory was applied, while for porosity, the evaluation employing the Barrett–Joyner–Halenda (BJH) method was used. The International Union of Pure and Applied Chemistry (IUPAC) defines six types of physical sorption isotherms (I–VI), where the shape analysis of isotherm gives information about the characterization of surface area and porosity of the material [44]. The obtained titanium dioxide isotherms are shown in Figure 1a.

The results revealed that the shape of the TiO_2_ isotherm is a combination of type II and type IV. Type II sorption isotherms are typical for non-porous or macroporous materials, for which unrestricted multilayer adsorption can occurs. In the isotherm, the characteristic bond (the knee) was found at low pressure, indicating the point at which the creation of a monolayer was completed. On the other hand, type IV is characteristic for mesoporous materials. As can be seen in Figure 1a, this isotherm possesses a characteristic feature of a hysteresis loop, which is the consequence of adsorbate condensation in TiO_2_ pores. The limiting adsorption at the high pressure close to 1.0 *p*/*p*^0^ results in a plateau of the isotherm, indicating the complete pore filling (according to the type IV isotherm model). However, this effect was not observed in the case of TiO_2_ since the material isotherm is a combination of both II and IV types. The shape of the hysteresis loop is correlated with pore size distribution and pore geometry. According to the IUPAC, four types of hysteresis loop shapes can be distinguished [44]. Based on the shape analysis of the TiO_2_ hysteresis loop (Figure 1a) one can assign it to the H3 shape, which means that the material structure consists of slit-shape mesopores. The result of this geometry can be explained by the phenomenon of the capillary condensation that occurs when titanium dioxide is exposed to the adsorbate.

According to the BET method of calculation, the investigated titanium dioxide is characterized by a surface area of 59.5 m^2^/g. Its structure is a combination of both mesopores (defined in the width range of 2–50 nm) and macropores (above 50 nm). Figure 1b shows the pore size distribution of TiO_2_. Based on the BJH method the average pore width of 6.2 nm and cumulative volume of pores of 0.117 m^3^/g were calculated. The obtained results indicate that the mesoporous structure of TiO_2_ dominates the macroporous structure.

### Characteristics of imines

**Scheme 1:**
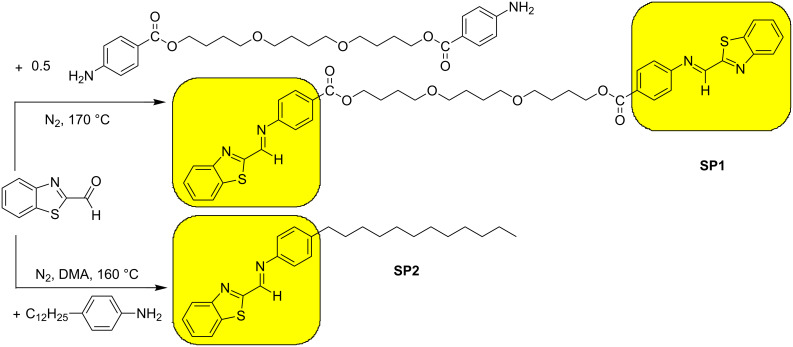
Synthetic route and chemical structure of SP1 and SP2 imines.

New imines, (*E*,*E*)-(butane-1,4-diyl)bis(oxybutane-4,1-diyl) bis(4-{[(benzo[*d*][1,3]thiazol-2-yl)methylidene]amino}benzoate) (SP1) and (*E*)-*N*-[(benzo[*d*][1,3]thiazol-2-yl)methylidene]-4-dodecylaniline (SP2), were obtained via high-temperature condensation. The synthetic route and chemical structure of both imines are presented in Scheme 1.

The strategy behind the synthesis of these two imine includes careful selection of benzothiazole aldehyde and two aromatic amines, 4-dodecylaniline and poly(butane-1,4-diol) bis(4-aminobenzoate). First, the synthesized compounds – aromatic Schiff bases – are known as thermally stable, and second, thanks to the plane structure, they will tend to adopt a column-like structure. In the case of the asymmetric molecule (SP2), it was expected to obtain a columnar structure in low-ordered mesophase, such as a nematic phase. Whereas, for the symmetric structure (SP1), an additional linkage between two benzothiazole units would cause some distortions and formation of a more ordered phase. Moreover, due to a significant number of donor heteroatoms, an interesting interaction with TiO_2_ was also expected.

In order to confirm the molecular structure of investigated SP1 and SP2 compounds, FT-MIR spectroscopy was carried out at room temperature. In the case of SP1, the most intensive bands in the IR spectrum are located at 1263 and 1099 cm^−1^ and correspond to stretching modes *ν*(C–O) within the RCOOR ester and C–O–C ether groups, respectively. Moreover, intensive and sharp bands at 1712 cm^−1^, characteristic for the *ν*(C=O) mode of carbonyl group, are observed. In the case of SP2, the most prominent bands are located in the high-wavenumber range at 2920 and 2847 cm^−1^ and correspond to asymmetric and symmetric stretching modes, *ν*_as_(CH_2_) and *ν*_s_(CH_2_), respectively, of CH_2_ groups from long alkyl chain. The sharp and medium intensity band characteristic of the *ν*(HC=N) stretching mode of the imine bond is observed at 1603 and 1617 cm^−1^ for SP1 and SP2, respectively, which confirms the presence of an azomethine group.

The characteristic bands of the benzothiazole group, the *ν*(C=N) and *ν*(C–S) modes, are located at about 1586 and 697 cm^−1^ in the case of SP1 and at 1585 and 689 cm^−1^ in case of SP2, respectively, which is in good agreement with the literature [45]. Several bands of small intensity assigned to the stretching modes of aromatic rings, *ν*(C–H)_ar_ modes (also from benzothiazole), can be observed in the spectral range of 3120–3000 cm^−1^, whereas the *ν*(C=C)_ar_ modes are located between 1600 and 1500 cm^−1^. In turn, the in-plane bending mode δ(CH)_ar_ is observed at 1013 cm^−1^ for both compounds. The out-of-plane bending modes γ(CH)_ar_ are observed at 854, 802 and 758 cm^−1^ for SP1 and at 833, 799 and 759 for SP2. Overall, the spectroscopic results showed all expected bands characteristic for the organic functional groups of the studied substances in the IR spectra.

**Figure 2:**
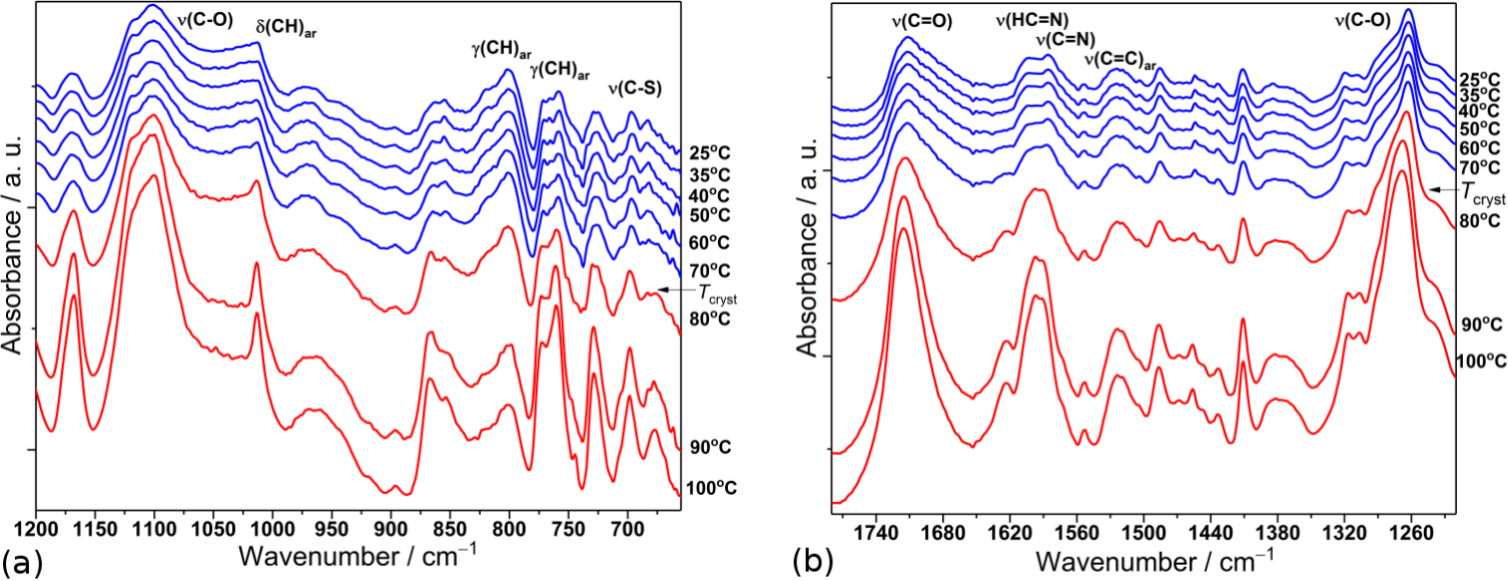
Temperature evolution of the FTIR spectrum of SP1 imine obtained during heating in two spectral ranges: 1200–650 cm^−1^ (a) and between 1780–1220 cm^−1^ (b).

The FT-MIR spectra of SP1 obtained at different temperatures during heating in two spectral ranges are presented in Figure 2.

In temperature range between 25 and 70 °C, the IR bands present no change. Moreover, the small, broad signal peaks suggest the presence of a vitreous state. When heated above 75 °C, the intensities of many bands, i.e., at 1712, 1586, 1263, 1102 and 758 cm^−1^, associated to *ν*(C=O), *ν*(C=N), two *ν*(C–O) and γ(CH)_ar_ modes, respectively, became sharper, more intense and narrower as the conversion into crystalline state increased. In turn, upon cooling from 100 to −123 °C we did not detect any significant changes in the spectra, the IR bands remained sharp and well-defined in whole studied temperature range, thus no back-vitrification process was observed.

**Figure 3:**
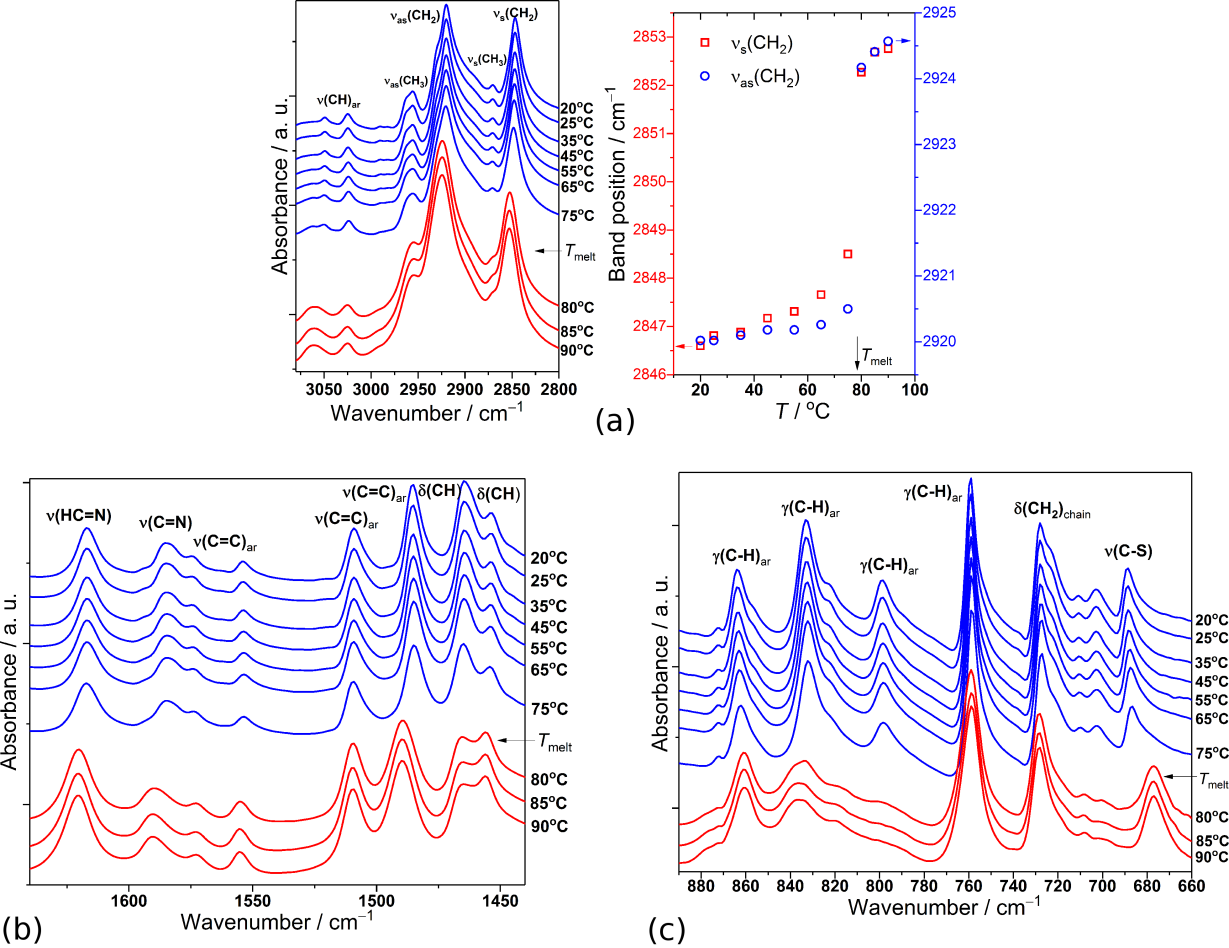
Temperature evolution of the FT-MIR spectrum of SP2 imine obtained during heating between: (a) 3080 and 2800 cm^−1^ (left) and temperature dependences of positions of the bands related to the alkyl chain (right), (b) between 1640 and 1440 cm^−1^ and (c) between 890 and 660 cm^−1^.

The temperature evolution of the IR spectrum registered in the high-wavenumber region (between 3080 and 2800 cm^−1^) upon heating from 20 to 90 °C for the SP2 sample is presented in Figure 3a.

As it can be seen in Figure 3a, apart from the low-intensity bands, the region above 3000 cm^−1^ can be related to the stretching modes *ν*(CH)_ar_ within aromatic rings. At room temperature, in the regions near 2920 and 2847 cm^−1^ appear for the characteristic intensive stretching vibrational modes, *ν*_as_(CH_2_) and *ν*_s_(CH_2_) respectively, of alkyl chains of the investigated compound. Their positions give evidence of highly ordered hydrocarbon chains with an all-trans conformation at this temperature. With increasing temperature, both bands shift significantly toward larger wavenumbers (Figure 3a). The band positions shift by about 5 cm^−1^ for *ν*_as_(CH_2_) and 6 cm^−1^ for *ν*_s_(CH_2_) modes above the isotropisation temperature. This shows the increase of conformational disorder and mobility in hydrocarbon chains as liquid phase is formed. Figure 3b illustrates the evolution of the IR spectrum of SP2 at different temperatures in the spectral range between 1640 and 1440 cm^−1^, where the bands characteristic of the internal vibrations within imine groups, thiazole and aromatic rings and alkyl chain occur. During the melting process, the positions of the IR bands assigned to the *ν*(HC=N)_imine_ and *ν*(C=N)_thiazole_ modes observed at 1617 and 1585 cm^−1^ shift toward larger wavenumbers of about 3 and 5 cm^−1^, respectively. Also, the band at 1485 cm^−1^ of *ν*(C=C)_ar_ mode in the benzothiazole group shifts toward larger wavenumbers by about 5 cm^−1^. Moreover, distinct changes in intensities of the bands connected to the scissoring and bending δ(CH) modes in alkyl chains located at 1465 and 1453 cm^−1^ are clearly visible. Some changes of the IR spectrum during the melting process can also be noticed in the lower wavenumber range (below 900 cm^−1^) as presented in Figure 3c. The shifting of the *ν*(C–S) mode toward lower wavenumbers by 12 cm^−1^ and the large change of intensity of the γ(CH)_ar_ modes located at 833 and 799 cm^−1^ are especially peculiar. This proves that all the molecular groups such as imine, benzothiazole, aromatic rings as well as alkyl chains, being part of SP2, are involved in the transition into the isotropic state. It is also worth mentioning that no significant changes in the IR spectra of SP2 are observed at temperatures between 20 to 75 °C, and therefore from the IR spectroscopy point of view, the presence of only one crystalline phase can be assumed.

**Figure 4:**
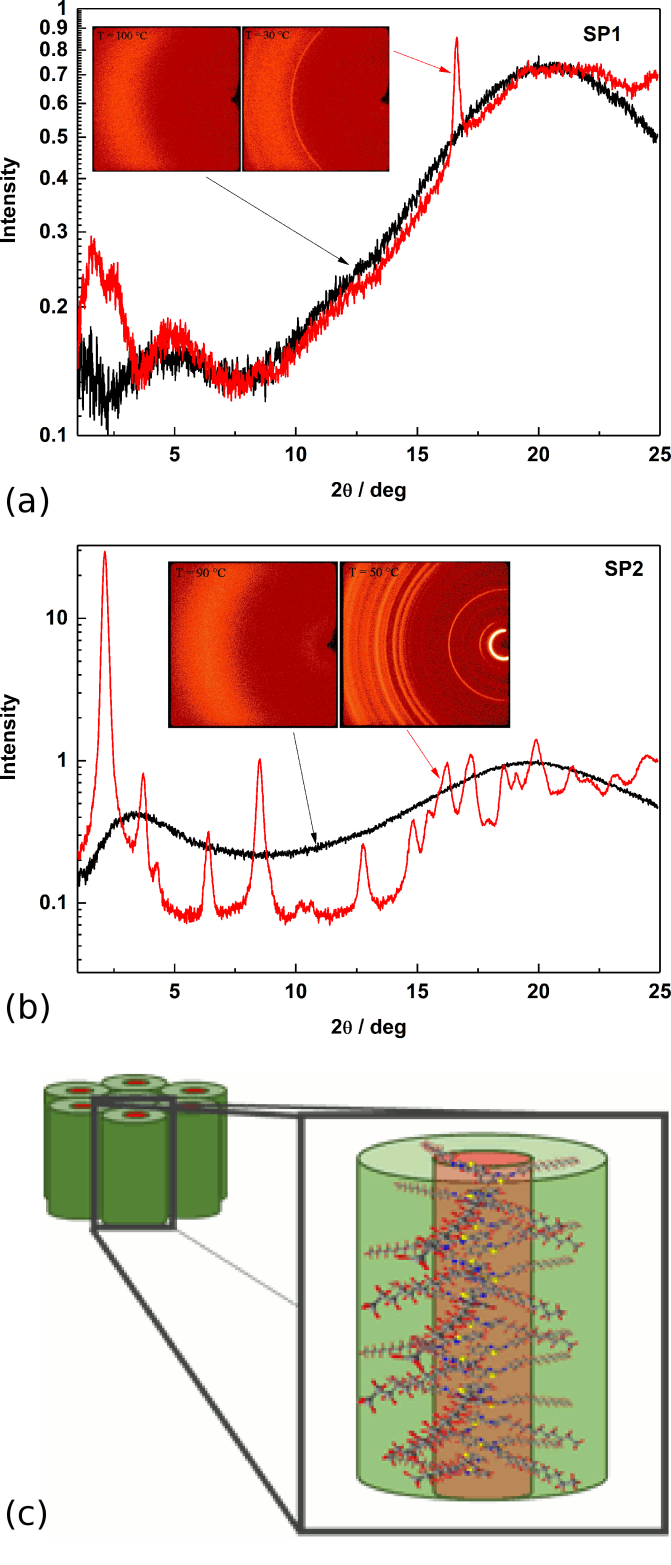
Intensity of X-ray signal vs diffraction angle, obtained by integration of 2D patterns over azimuthal angle and 2D XRD patterns obtained for: (a) SP1 imine (at 30 and 100 °C), (b) SP2 imine (at 50 and 90 °C) and (c) visualization of hexagonal packing of SP2 columns.

The XRD studies performed in the broad diffraction angle range of SP1 and SP2 imines exhibited quite different behaviors. In detail, the wide-angle X-ray diffractogram at 30 °C for SP1 imine (see Figure 4) revealed several signals with intraplanar spacings at 56.6 Å, 35.5 Å, 18.8 Å, 5.3 Å and 4.3 Å, of which the first three correspond to the planes (100), (110) and (300) of columnar organization, respectively.

The presence of a sharp signal and a broad halo at higher 2θ values could give the impression of a short-range in-plane positional order, similar to that reported by Repasky et al. [46] for alkyl-substituted dibenzo[*fg,op*]tetracenes in B_hex_ phase and not, as one could assume, more-ordered SmB phase like for a symmetrical azomethine with biphenyl mesogenic groups described by us in our previous paper [47]. The measurement performed at 100 °C for SP1 imine (isotropic phase) displays only a broad signal at 4.4 Å.

The diffraction pattern for SP2 at 50 °C, suggested a hexagonal columnar crystalline phase (Col_hk_), as evident from the high number of sharp reflections (Figure 4). As a matter of a fact, the reflections located at 2θ = 2.2°, 3.7°, 4.3° and 6.4°, corresponding to *d* = 41.1 Å, 23.9 Å, 20.4 Å and 13.8 Å, exhibited d-spacings in the ratio 1:1/√2:1/2:1/3 and could be assigned to (100), (110), (200) and (300) planes, respectively. The presence of other numerous sharp reflections gives evidence of the crystalline nature of the SP2 compound. On the other hand above the melting temperature (90 °C) all sharp signals disappeared leaving only a broad halo at 4.6 Å.

**Figure 5:**
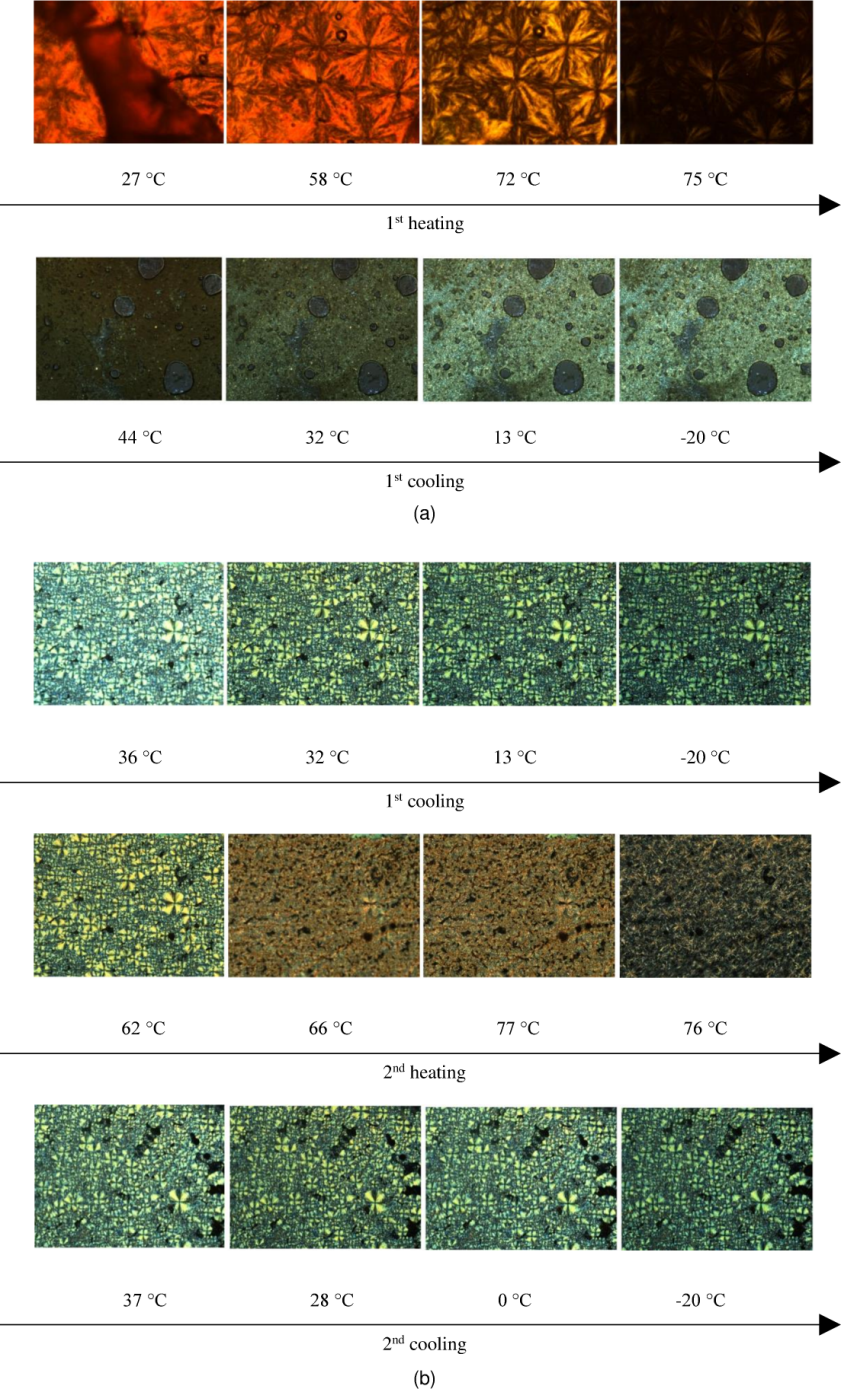
Polarizing optical microscopy (POM) images for: (a) SP1 taken during 1^st^ heating (at 27, 58, 72, 75 °C) and 1^st^ cooling (at 44, 32, 13 and −20 °C), respectively and (b) SP2 taken during 1^st^ cooling (at 36, 32, 0 and −20 °C), 2^nd^ heating (at 62, 66, 77, 76 °C) and 2^nd^ cooling (at 37, 28, 0 and −20 °C), respectively.

Both compounds were widely investigated by differential scanning calorimetry (DSC) and polarizing optical microscopy (POM) to describe its thermal behavior taking into consideration the different symmetry of SP1 and SP2. POM images registered at heating and cooling for SP1 and SP2 are shown in Figure 5.

**Figure 6:**
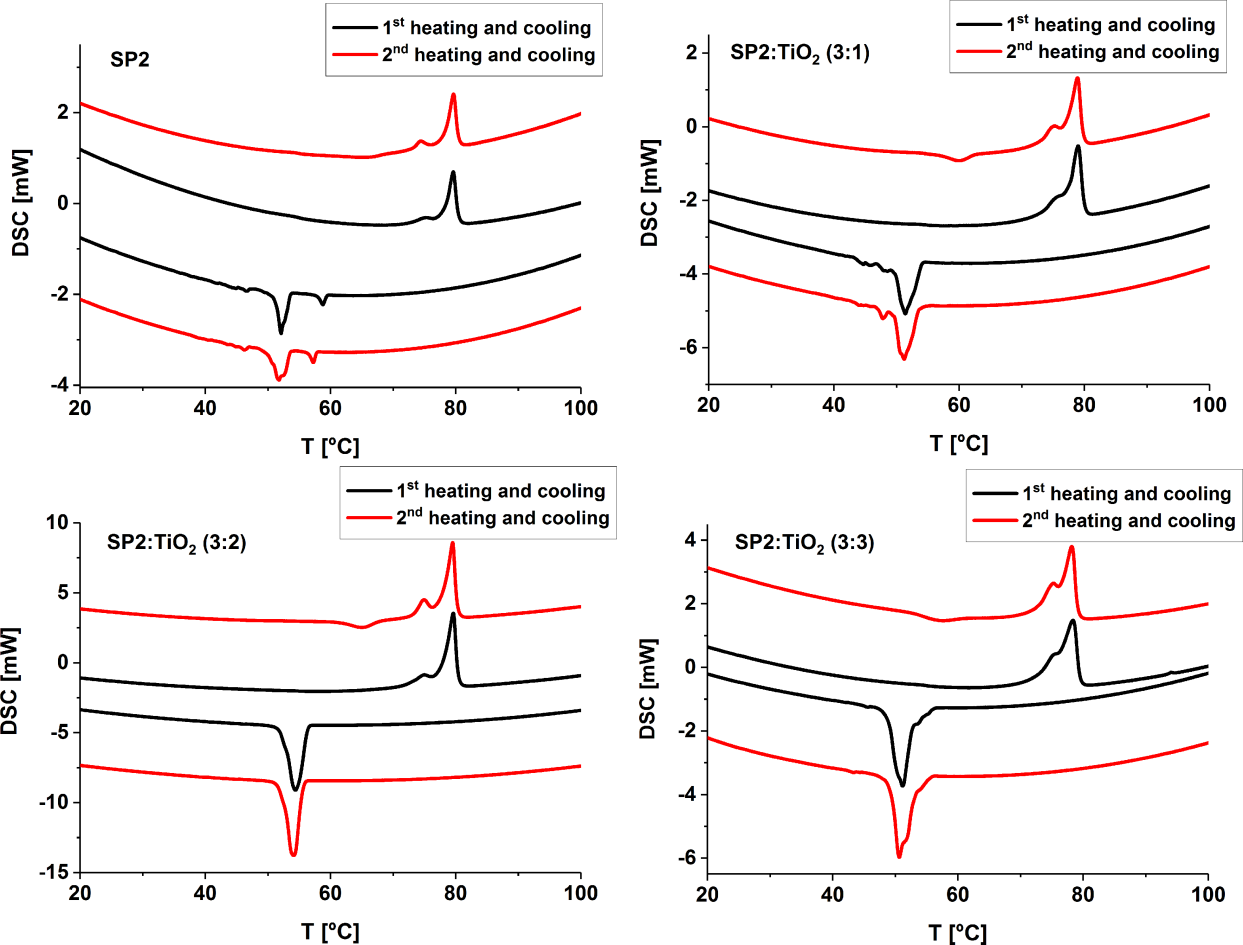
Differential scanning calorimetry (DSC) curves of the SP2 and SP2:TiO_2_ mixtures registered at the 1^st^ and 2^nd^ heating/cooling cycles with a rate of 10 °C/min.

Asymmetrical imine SP2 exhibited a higher melting point (mp 78.39 °C) than symmetrical SP1 (mp 66.31 °C). DSC curves of SP2 exhibited two peaks (see Figure 6), where the most intense one is attributed to the phase transition into isotropic liquid.

However, the less intense peak at lower temperature is connected with the transition between two crystalline phases. For SP1 there is only one texture and one peak at 66.31 °C in the DSC curve on heating with the rate of 10 °C/min. Additionally, SP1 was measured during heating and cooling with increasing rates, because at a rate of 10 °C/min, no anomaly upon cooling was noticed. It was found that the cycles with higher scanning rates differ from those previously reported and at a rate of 30 °C/min, a visible anomaly during cooling was registered (*T*_cooling_ = 32.28 °C, Δ*H* = −4.14 J/g).

The electrochemical properties of SP1 and SP2 imines were investigated in CH_2_Cl_2_ solution by means of cyclic voltammetry (CV). To estimate ionization potential and electron affinity (HOMO and LUMO energies) of the materials, the IP of ferrocene of −5.1 eV was assumed [48]. Table 1 contains calculated HOMO and LUMO levels, and the values for the electrochemical energy band gap (*E*_g_).

**Table 1:** Electrochemical and energy level parameters of investigated compounds and mixtures.

Code	*E*_red_ (onset, CV), [V]	*E*_ox_ (onset, CV), [V]	*E*_g_^CV a^ [eV]	HOMO^b^ [eV]	LUMO^c^ [eV]

SP1	−1.51	0.84	2.35	−5.94	−3.59
SP2	−1.78	1.29	3.07	−6.39	−3.32
SP1:TiO_2_ (1:1)	−1.6	0.73	2.33	−5.83	−3.5
SP2:TiO_2_ (1:1)	−1.68	0.43	2.11	−5.53	−3.42
SP2:TiO_2_ (3:2)	−1.7	0.38	2.08	−5.48	−3.4
SP2:TiO_2_ (3:1)	−1.72	0.32	2.04	−5.42	−3.38
SP1:P3HT (1:1)	−1.65	0.35	2.00	−5.45	−3.45
SP2:P3HT (1:1)	−1.78	0.35	2.13	−5.45	−3.32
P3HT	−2.16	0.07	2.23	−5.17	−2.94
TiO_2_	−0.91	2.1	3.01	−7.2	−4.19

^a^*E*_g_^CV^ = *E*_ox_ (onset) − *E*_red1_ (onset); ^b^HOMO = −5.1 − *E*_ox_; ^c^LUMO = −5.1 − *E*_red_.

**Figure 7:**
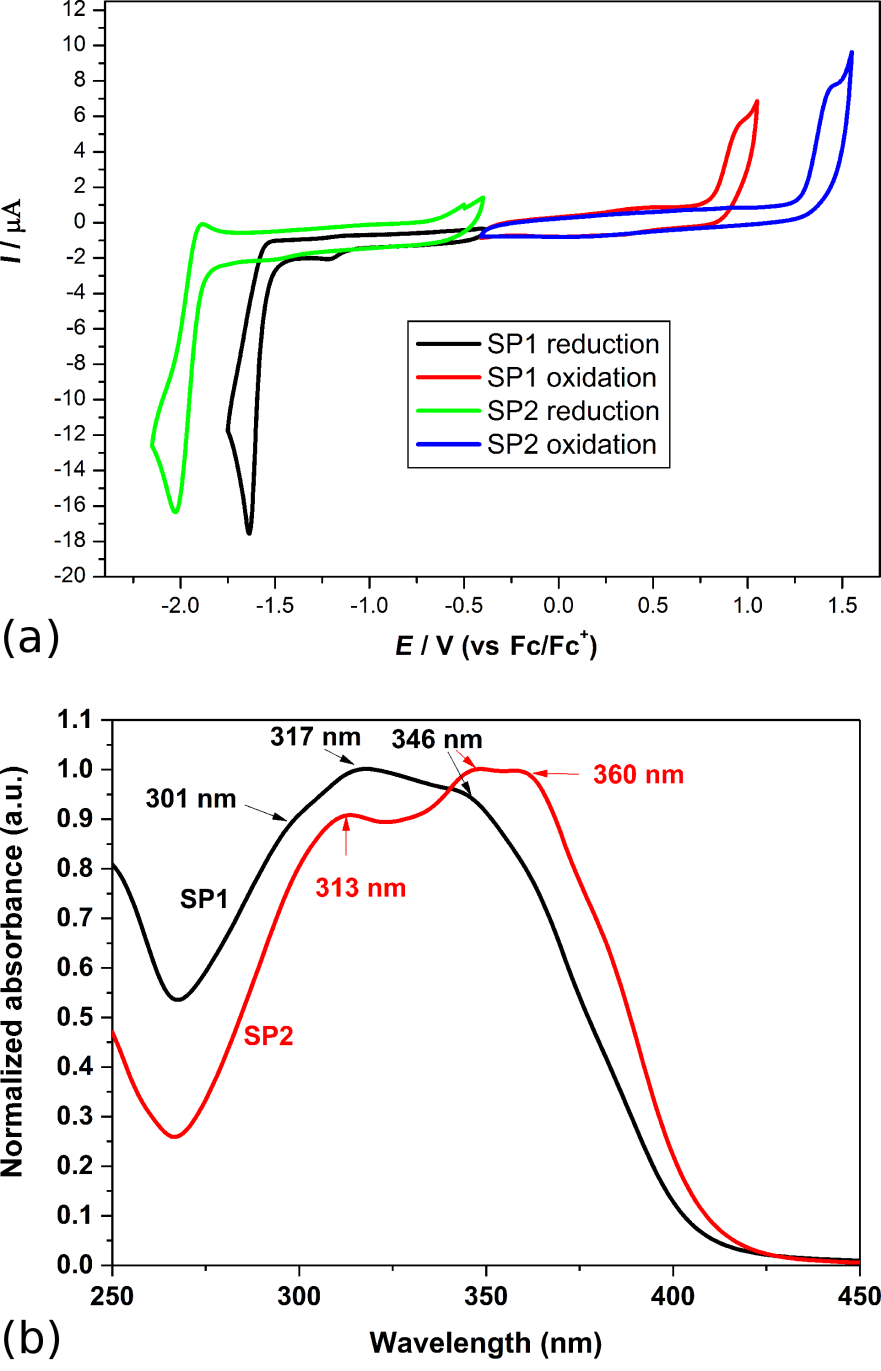
(a) Cyclic voltammograms for SP1 and SP2. CV sweep rate *ν* = 100 mV/s, 0.1 M Bu_4_NPF_6_ in CH_2_Cl_2_, (b) UV–vis spectra of both imines in chloroform solution.

The electrochemical properties of pure SP1 and SP2 in solution were examined. The behavior of both compounds during reduction and oxidation processes were investigated in order to determine the energy levels and band gaps. For SP1 imine, the peak onsets were as follows: *E*_red_ = −1.51 V and *E*_ox_ = 0.84 V. In the case of SP2 imine, similar processes occur significantly stronger as demonstrated by the energy values *E*_red_ = −1.78 V and *E*_ox_ = 1.29 V (see Figure 7a). It also affects the electrochemically determined band gaps, which are much larger for SP2 (3.07 eV) than for SP1 (2.35 eV). The direct influence of the ester groups from the SP1 molecule is obvious.

The UV–vis absorption spectrum of SP1 in chloroform solution revealed one main peak at 317 nm and two shoulder peaks at 301 nm and 346 nm (see Figure 7b). While for SP2, three main absorption peaks at 313, 346 and 360 nm were found. The *E*_g_ values obtained with the two different methods, UV–vis and CV, were compared, and the results for SP2 are in good agreement (*E*_g_^UV–vis^ = 2.95 eV, *E*_g_^CV^ = 3.07 eV). However, for SP1, some differences were observed (*E*_g_^UV–vis^ = 3.03 eV, *E*_g_^CV^ = 2.35 eV). It is known that the energy differences obtained by these methods are typically not identical and can be as large as 1 eV. Further studies are needed to clarify this issue.

### Improved properties of TiO_2_:imine composites

**Figure 8:**
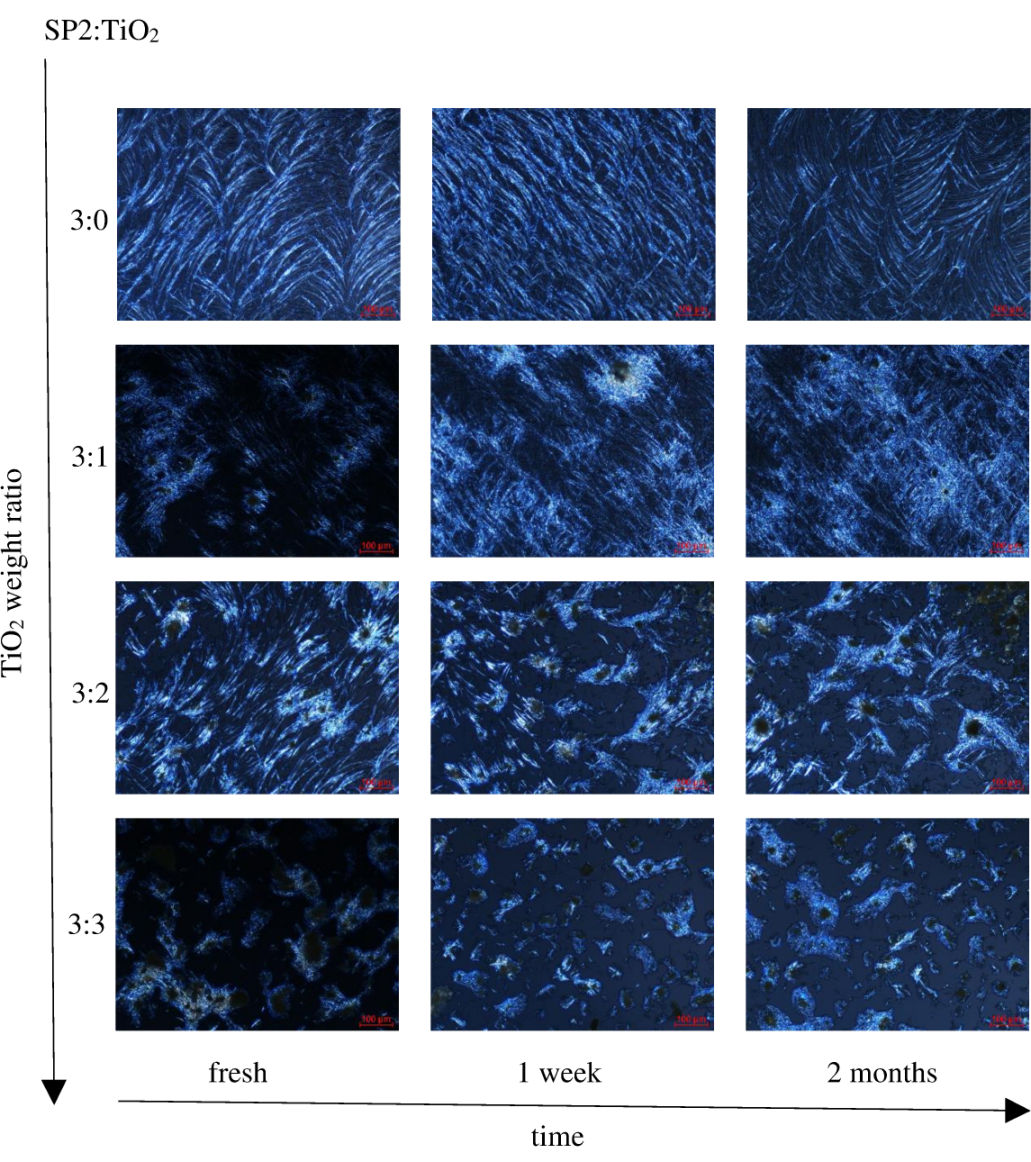
Polarizing optical microscopy (POM) textures of SP2:TiO_2_ mixtures with weight ratios: 3:0 w/w, 3:1 w/w, 3:2 w/w and 3:3 w/w observed at about 22 °C (from top to bottom, respectively) for fresh mixtures after 1 week of preparation and after 2 months of preparation (from left to right side, respectively).

The influence of the amount of TiO_2_, time and number of heating/cooling cycles was examined for SP1 and SP2 imines mixed with TiO_2_. Figure 8 shows POM textures of SP2 imine and SP2:TiO_2_ mixtures with different weight ratios 3:3, 3:2, 3:1, 3:0, taken after different periods of time under standard laboratory conditions (humidity about 50%, temperature about 24 °C). The investigated samples on glass substrate were kept in the laboratory without any protection from this environment.

**Figure 9:**
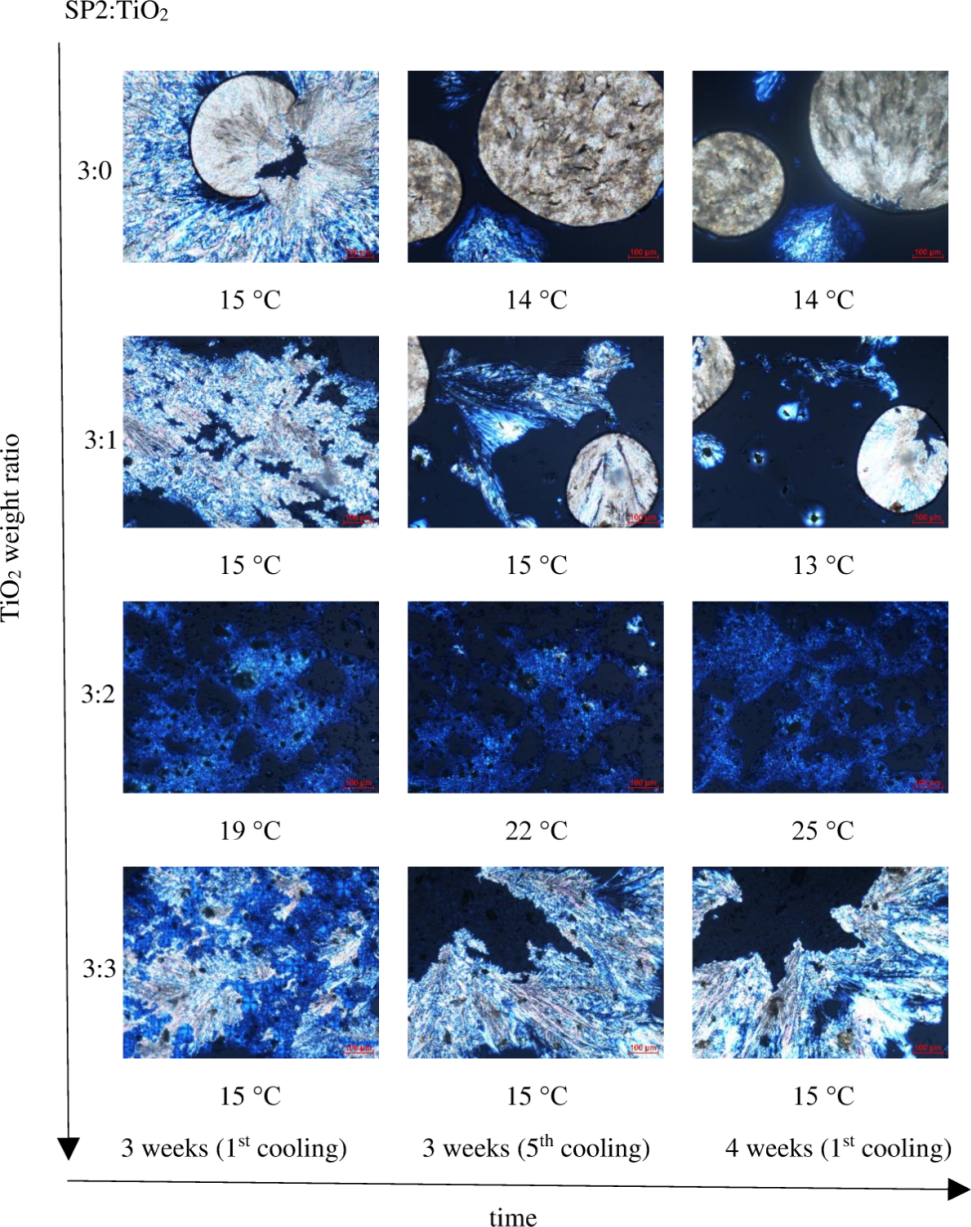
Polarizing optical microscopy (POM) textures of SP2:TiO_2_ mixtures with weight ratio: 3:0 w/w (at about 15 °C), 3:1 w/w (at 15 °C and 13 °C (right side)), 3:2 w/w (at 19, 22 and 24 °C, respectively) and 3:3 w/w (at about 15 °C), observed during cooling of the sample after 3 weeks (left and middle sides images) and after 4 weeks (right side images).

It can be seen that a layer of pure SP2 imine is the most homogenous and smaller domains are formed on the glass surface with increasing amount of TiO_2_. Furthermore, the textures of the SP2 layer obtained after evaporation of the solvent (chloroform) differ from that for pure SP2. However, the next cycles of heating and cooling caused changes in textures. As it is presented in Figure 9, after recrystallization from isotropic liquid during cooling, different textures appeared and become stabilized after a few heating–cooling cycles. Only for the SP2:TiO_2_ mixture in the ratio of 3:2 w/w were the textures always the same throughout entire process. For SP2:TiO_2_ 3:0 w/w and 3:1 w/w mixtures, the isotropization temperature resulted in a change in the image. In general, the layers become more stable with increasing amount of TiO_2_.

DSC curves (Figure 6) for SP2:TiO_2_ mixtures with different concentrations of TiO_2_ were measured during heating and cooling at a rate of 10 °C/min. The phase transition temperatures changed slightly with increase in the concentration of TiO_2_ (see Table 2).

**Table 2:** Transition temperatures and associated enthalpy changes of SP2 imine and its mixture with TiO_2_ in the various ratios upon 1^st^, 2^nd^ heating and cooling cycles at a rate of 10 °C/min.

Code	Transition temperatures (°C) (Δ*H*, *J*/*g*)	

	Heating runs	Cooling runs

1^st^ cycle		

SP2	73.96 (3.51), 78.39 (51.62)	59.30 (−7.17), 53.15 (−49.86)
SP2:TiO_2_ (3:1)	74.53 (1.32), 77.56 (29.38)	53.85 (−37.10)
SP2:TiO_2_ (3:2)	73.68 (2.31), 77.88 (36.68)	56.31 (−62.62)
SP2:TiO_2_ (3:3)	74.59 (0.51), 76.46 (17.44)	52.91 (−69.82)

2^nd^ cycle		

SP2	73.59 (5.38), 78.30 (54.38)	57.75 (−7.27), 53.49 (−52.72)
SP2:TiO_2_ (3:1)	56.69 (−10.62), 73.83 (1.44), 77.41 (21.77)	53.36 (−39.73)
SP2:TiO_2_ (3:2)	62.43 (−10.07), 73.58(5.70), 77.92 (30.64)	55.51 (−59.72)
SP2:TiO_2_ (3:3)	53.90 (−9.16), 73.63 (3.08), 76.62 (14.44)	53.21 (−61.64)

It also turned out that titanium dioxide affects the melting temperature of SP1. For example, the mixture SP1:TiO_2_ (3:2 w/w) exhibited a melting point which is about 8 °C higher than the pure SP1. Unfortunately, for this mixture it was not possible to achieve textures during cooling and there was no anomaly on the DSC curve at cooling. This may be because the thickness of this substance is too high or due to the very slow crystallization from isotropic liquid. Furthermore, both the imines (SP1 and SP2) aggregate into small islands after evaporation of solvent (not shown here). After 3 months of being kept in air atmosphere, both SP2 and their mixtures with TiO_2_ remained unchanged, which makes them an interesting material for organic devices, e.g., photovoltaic applications.

**Figure 10:**
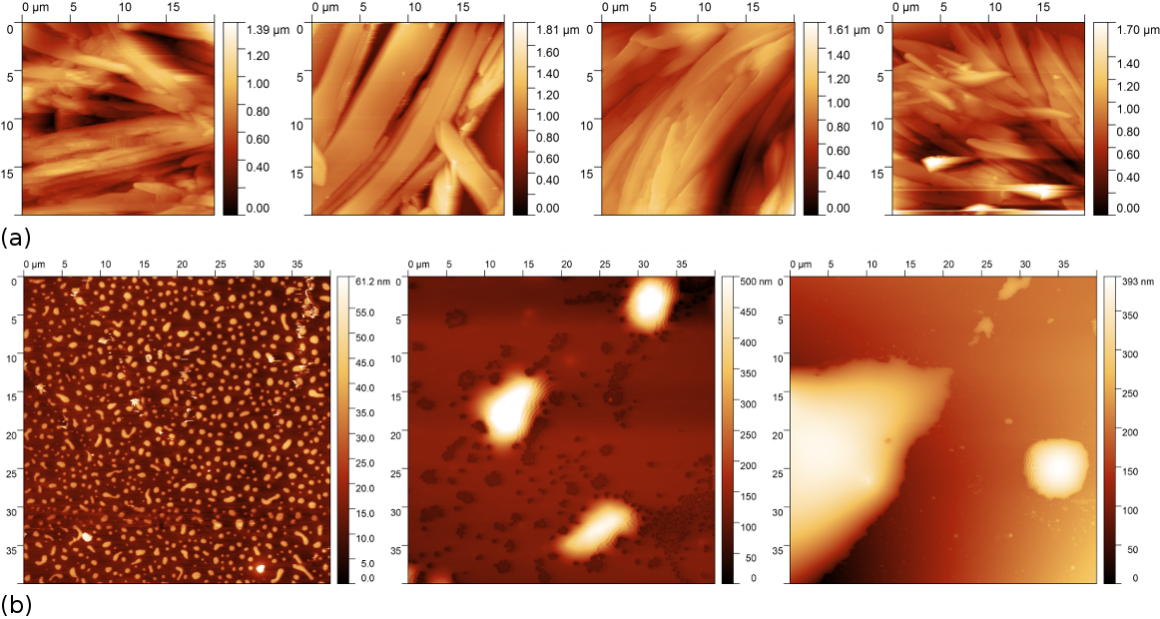
AFM images of: (a) SP2, SP2:TiO_2_ (3:1 w/w), SP2:TiO_2_ (3:2 w/w), SP2:TiO_2_ (3:3 w/w), and (b) SP1, SP1:TiO_2_ (3:2 w/w), TiO_2_, respectively from left to right.

In the AFM experiments the influence of the TiO_2_ and the chemical structure of the imines with the benzothiazole core on the surface morphology of the materials was investigated. Thin layers of SP2 and SP2:TiO_2_ composites in various weight ratios were prepared by dropping the solution on a glass substrate and annealed on the hot plate at 60 °C to evaporate the solvent (chlorobenzene). Since layers prepared from SP1 and TiO_2_ using the same technique turned out to be too rough to be measured with AFM, these samples were prepared with the spin coating technique (1000 rpm, 40 s). As one can see in Figure 10a, the topography of pure SP2 imine and SP2:TiO_2_ composites in each case reveals quite large crystallites randomly distributed on the glass surface, and no significant difference between pure SP2 and SP2 doped with TiO_2_ was noticed.

On the other hand pure SP1, TiO_2_ and SP1:TiO_2_ (3:2 w/w) do not show good film forming properties on the glass surface (see Figure 10b). This is due to the incompatible nature of hydrophobic compounds on hydrophilic glass. It can be also seen that a uniform layer for pure SP1 was formed. On the other hand, pure TiO_2_ forms agglomerates of much larger areas than SP1. The image obtained for the SP1:TiO_2_ mixture shows that TiO_2_ interacted with SP1 to form a completely different layer than for unmixed substances. The higher topographies observed for SP1:TiO_2_ suggest higher TiO_2_ concentrations.

The value of roughness (Rms) varied significantly depending on the measured layer. For SP1, the imine Rms value was 9.06 nm whereas for SP1:TiO_2_ (3:2 w/w) it was 78.21 nm. Moreover, for SP2, imine Rms was 175 nm while for SP2:TiO_2_ composites, the value of Rms decreases with increasing amount of titanium dioxide: 255.5 nm (3:1 w/w), 196.9 nm (3:2 w/w) and 149.7 nm (3:3 w/w). For pure titanium dioxide, the Rms was 45.26 nm. The most desired surface morphology would be a layer composed of TiO_2_ nanoparticles covered with SP1/SP2 molecules like in a dye-sensitized solar cell model system [49].

**Figure 11:**
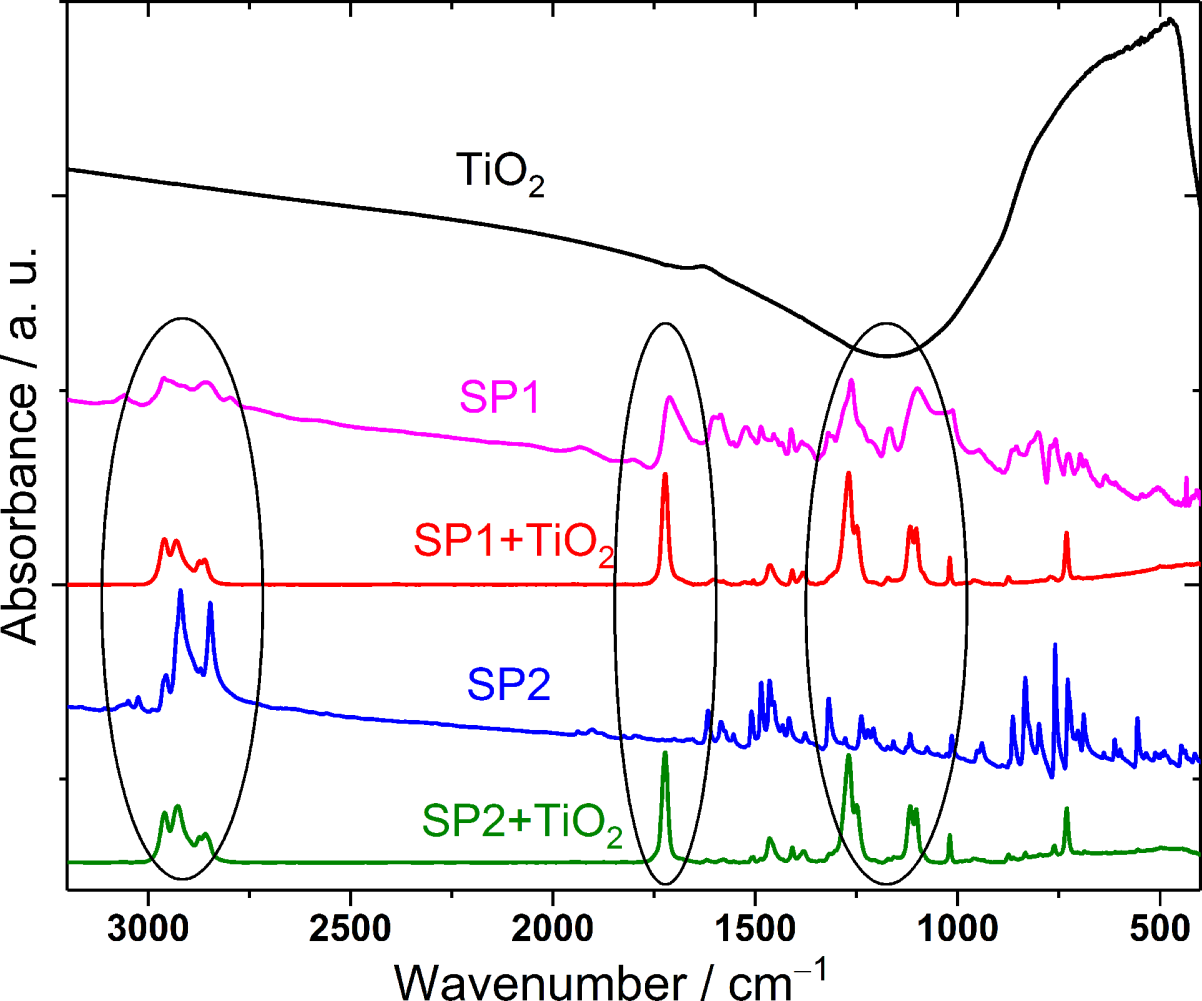
Comparison of the FT-MIR spectra of the SP1:TiO_2_ (3:2 w/w) and SP2:TiO_2_ (3:2 w/w) mixtures and their components between 3200 and 400 cm^−1^.

The influence of TiO_2_ on the molecular behavior and structure of both SP1 and SP2 benzothiazole derivatives has been also studied by FT-MIR spectroscopy, and the results are presented in Figure 11.

**Figure 12:**
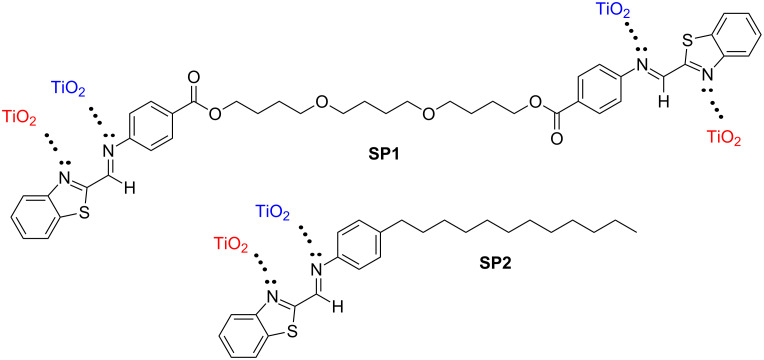
Interactions between TiO_2_ and SP1 or SP2.

The IR spectra of the SP1:TiO_2_ and SP2:TiO_2_ mixtures and their pure components, TiO_2_, SP1 and SP2, were obtained at room temperature. The most dominant and broad band in the spectrum of pure TiO_2_ in anatase form is located in the low wavenumber region below 1000 cm^−1^ and related to the *ν*(Ti–O) stretching mode. Interestingly, the FT-MIR spectra of SP1 and SP2 blended with TiO_2_ are basically the same, but compared to pure components, exhibit large differences. Both the positions and intensities of the selected bands are changed. The most intense and sharp bands in the spectra of the mixtures are observed at 1723, 1269 and 1117 cm^−1^. The first one corresponds the *ν*(C=O) mode of carbonyl group. The other two bands are also characteristic and most likely connected to the *ν*(C–O) modes within the ester group. Both IR spectra also contain bands showing the presence of alkyl chains at 2930 and 2858 cm^−1^ associated with the stretching modes *ν*_as_(CH_2_) and *ν*_s_(CH_2_), respectively, as well as bands at 1464 and 1382 cm^−1^ associated with scissoring and rocking bending modes δ(CH), respectively. Moreover, the lack of the IR bands connected to double C=C or triple C≡CH bonds indicate that the SP1 and SP2 mixtures with TiO_2_ signals from unsaturated hydrocarbon chains or aromatic rings are not visible. The observed changes confirmed the significant influence of titanium dioxide on the structural properties of both investigated compounds. It can be explained by the interactions of oxygen vacancies existing on the TiO_2_ surface with SP1 as well as SP2 occurring mainly in the same way. Similar interactions of SP1 and SP2 with TiO_2_ can be explained by the presence in both compounds aromatic imine moieties in thiazole ring and formation of the ≥N–Ti bond in imine:TiO_2_ composites (see Figure 12). Moreover, interactions between TiO_2_ and imine bonds (–N=CH–) cannot be excluded (see Figure 12). Jayabharathi et al. [1] observed analog interactions between TiO_2_ and imidazoles.

**Figure 13:**
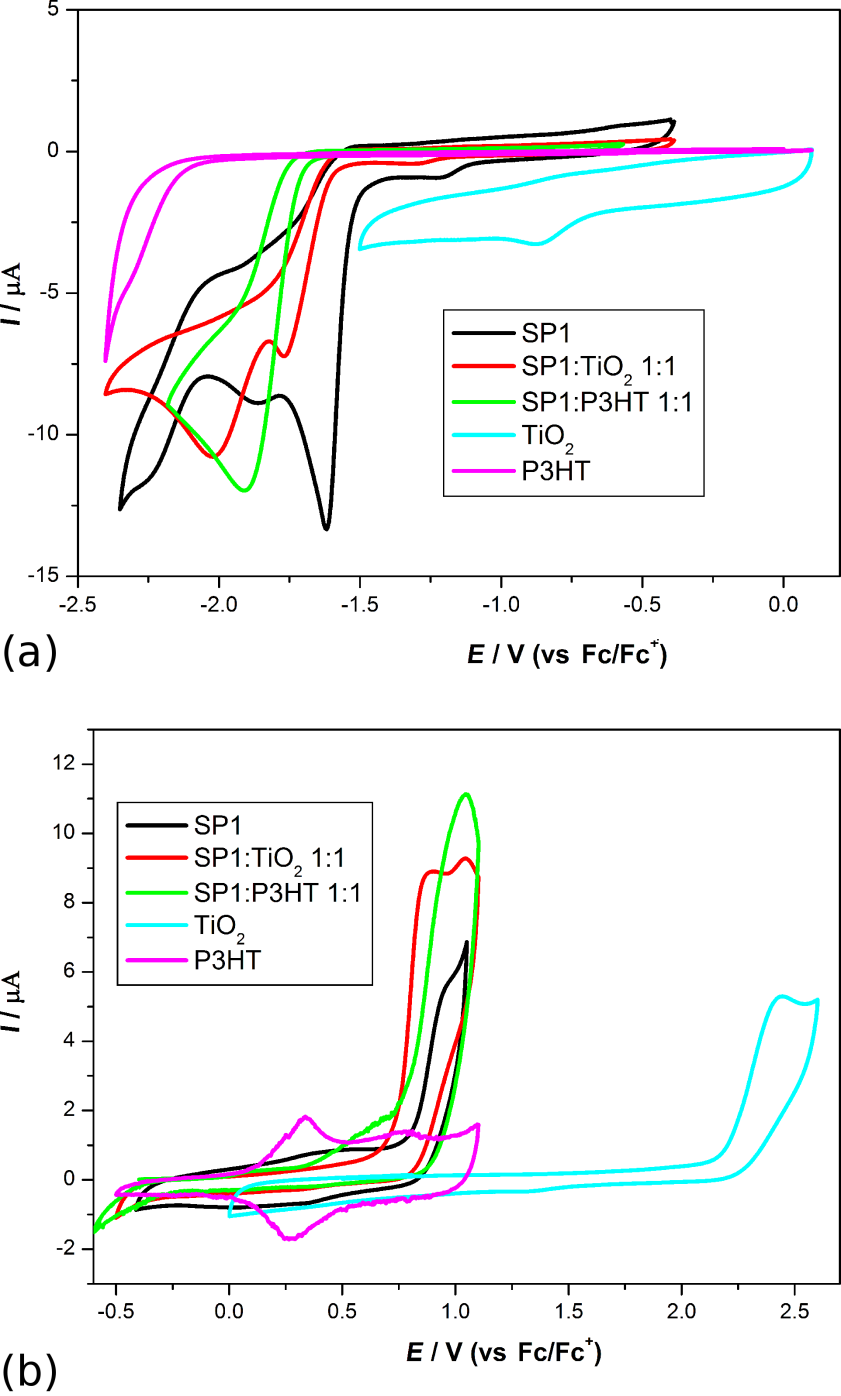
Cyclic voltammograms for SP1 and its mixtures with TiO_2_ and P3HT during reduction (a) and oxidation (b). CV sweep rate *ν* = 100 mV/s, 0.1 M Bu_4_NPF_6_ in CH_2_Cl_2_.

Next, we carried out a series of CV measurements for imine:TiO_2_ and imine:P3HT mixtures. As can be seen in Figure 13a and in Table 1, the addition of titanium dioxide and P3HT to SP1 at a weight ratio of 1:1 lowered the reduction peak onset to −1.6 V and −1.65 V, respectively.

However, in both cases mentioned above, additives shifted the electrochemical response of SP1 but no Faradic process was observed, which could be associated with TiO_2_ or P3HT reduction (in this potential region). On the other hand, as it can be seen in Figure 13b for the SP1:TiO_2_ (1:1 w/w) mixture, two well-exhibited oxidation waves were registered at a potential of 90 mV, which is lower as compared with the value obtained for SP1 without TiO_2_. Finally, the compound with the P3HT (1:1 w/w) mixture was prepared and examined during positive potential sweep. The first oxidation peak is broad with an onset as high as 0.35 V (probably process dominated by P3HT oxidation), followed by a second peak with an onset of 0.85 V (thus, as potential similar to oxidation of pure SP1). A similar behavior was observed in the case of the SP2:P3HT (1:1 w/w) mixture: the shift in reduction properties for SP2 is almost negligible, while in the positive region, the voltammogram is dominated by p-doping of the polymer.

**Figure 14:**
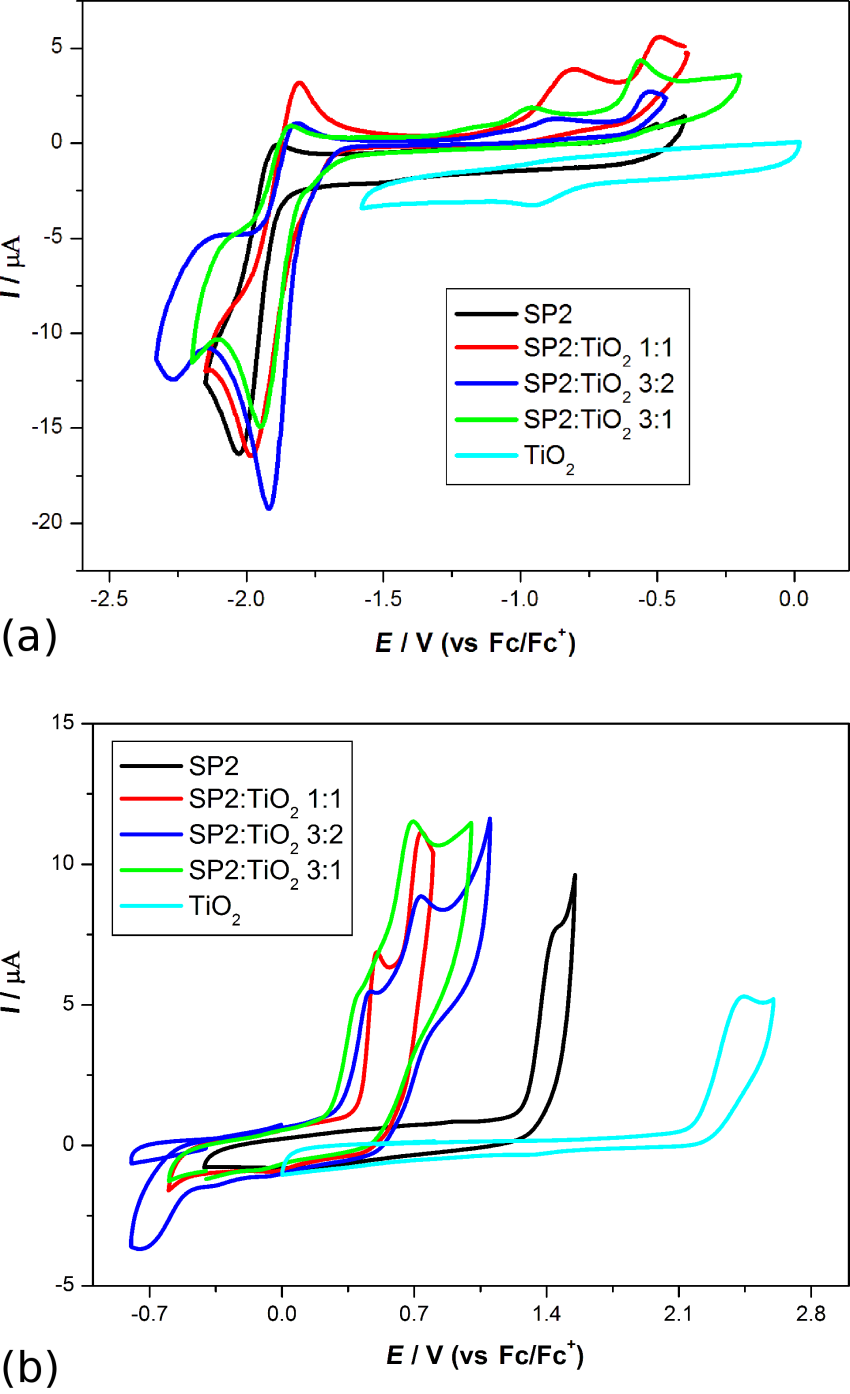
Cyclic voltammograms for TiO_2_, SP2 and its mixtures with TiO_2_ in various weight ratios during reduction (a) and oxidation (b). CV sweep rate *ν* = 100 mV/s, 0.1 M Bu_4_NPF_6_ in CH_2_Cl_2_.

Moreover, we carried out the series of measurements for mixtures of SP2 and TiO_2_ (in various proportions) as is presented in Figure 14.

In each case we observed a decrease in the reduction potential compared to pure SP1 compound. It is worth noting that the influence of TiO_2_ on properties of the investigated derivative during oxidation is even more significant – i.e. the peak onset is 0.8 V lowered independently from the ratio of compound: TiO_2_. Moreover, this becomes a two-step process with well-exhibited maxima. This implies that TiO_2_ provides not only a larger surface area for sensitizer adsorption and good electron transport [50], but can also shift the dye energy levels by intermolecular interaction. However, the quantity of these interactions is hard to predict a priori.

**Figure 15:**
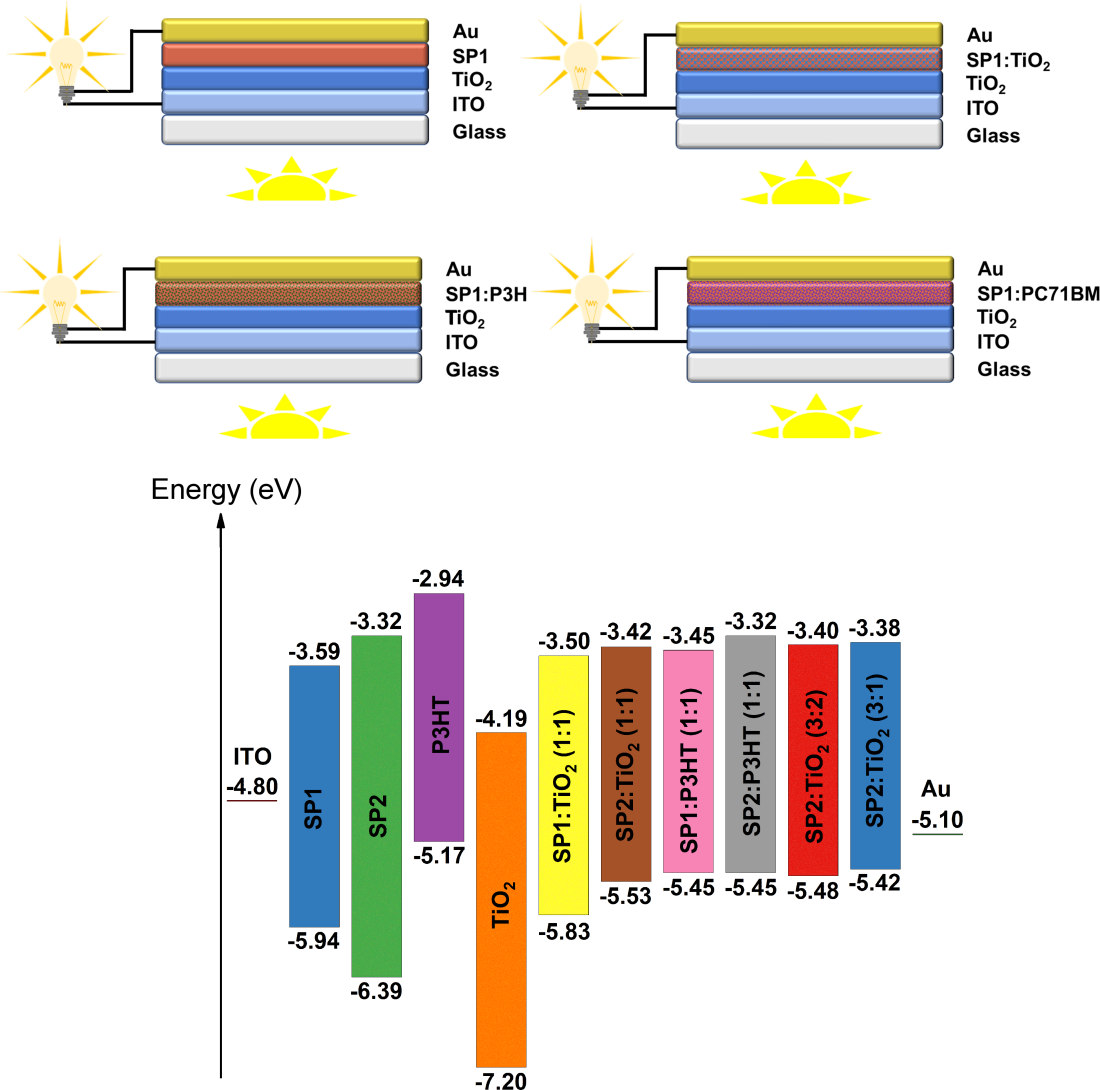
Architecture of the investigated devices along with the energy level diagram of donor and acceptors based on CV experiments and the work function of the different electrodes.

Finally, we built four organic devices with TiO_2_ and active layers based on SP1 used as a representative example of an air-stable organic compound. As reference sample, a device with P3HT was constructed. The architecture of the investigated devices, together with energy level diagram of donor and acceptors based on the CV experiments, as well as the work function of both electrodes, are presented in Figure 15.

It turned out that all investigated devices based on SP1 imine, independent of the active layer composition, exhibited very low current values on the order 10^−7^ mA. The reference device ITO/TiO_2_/P3HT/Au exhibited a higher value of current (≈0.02 mA). This behavior can be explained by inefficient light absorption of the active layer, a narrow range of maximum absorption bands (from 260 nm to 420 nm) and a high *E*_g_ (2.35 eV for SP1 and 3.07 eV for SP2) for both investigated imines. The turn-on voltage of the ITO/TiO_2_/SP1/Au device was observed at about 2.5 V at room temperature under illumination. The biggest increase of current at about 10 V was found for the device with SP1:PC_71_BM (1:1 w/w), where PC_71_BM: [6,6]-phenyl C_71_-butyric acid methyl ester. The ITO/TiO_2_/SP1:TiO_2_/Au and ITO/TiO_2_/SP1:P3HT/Au (P3HT:poly(3-hexylthiophene)) devices exhibited similar behavior – an increase of current at about 5 V. Our results showed that the investigated SP1 imine exhibited response under ≈88 mW/cm^2^ illumination.

**Figure 16:**
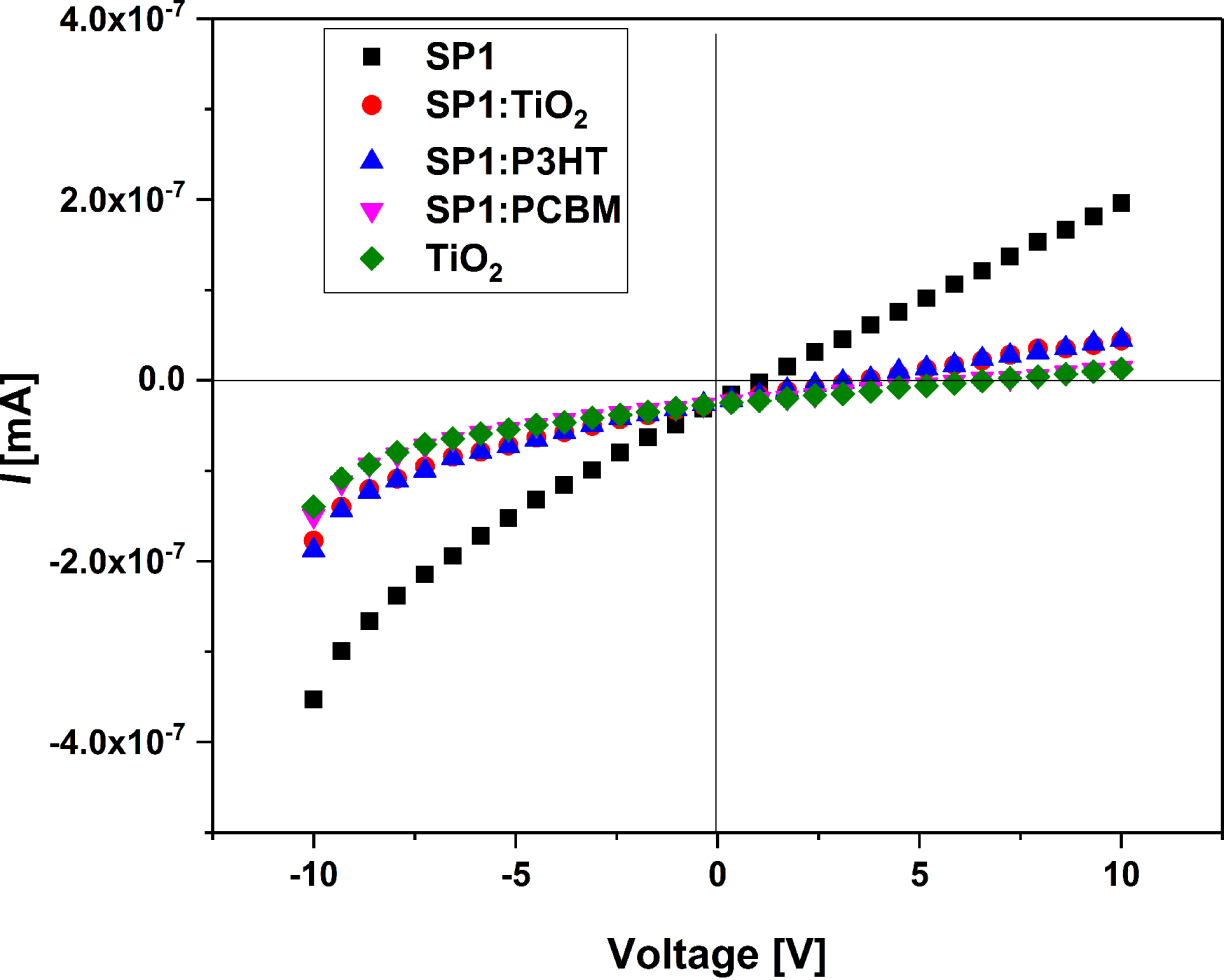
*I*–*V* curves of the ITO/TiO_2_/active layer/Au systems under 88 mW/cm^2^ illumination: I–IV characteristics.

The effect on the *I*–*V* characteristics suggested differences in the planarity and conformation of the SP1 imine in the pure imine film or in the mixture with TiO_2_, PCBM and P3HT. Our results showed that investigated SP1 imine exhibited response under 88 mW/cm^2^ illumination (see Figure 16), however, the current architecture of the device is more likely to be used as a photodiode (*I*–*V* characteristics at first (I) and third (III) part of spectrum) than for solar cells (small effect under illumination at fourth (IV) part of current–voltage characteristics). However, additional work is required to improve the electrical properties of the investigated devices.

## Conclusion

In this work we characterize of the interaction between TiO_2_ in anatase form and new symmetrical and asymmetrical imines. The structural, thermal and electrochemical properties of new synthesized imines, (*E*,*E*)-(butane-1,4-diyl)bis(oxybutane-4,1-diyl) bis(4-{[(benzo[*d*][1,3]thiazol-2-yl)methylidene]amino}benzoate) and (*E*)-*N*-[(benzo[*d*][1,3]thiazol-2-yl)methylidene]-4-dodecylaniline, have been investigated in detail. The interaction of SP1 and SP2 imines with TiO_2_ was found between the nitrogen atom from the thiazole ring and the nitrogen atom from the created imines with TiO_2_ particles. The symmetry of the investigated imines has an impact on the observed results of the created imine:TiO_2_ composites. Clearly, the investigated titanium oxide changed the properties of both imines in the following ways:

- weight ratio of TiO_2_ weakly affected the phase transition temperatures of both imines
- addition of TiO_2_ to SP2 imine significantly changed the POM texture of imine
- values of the energy gap and HOMO–LUMO levels of both imines decreased with increasing addition of titanium dioxide
- TiO_2_ has an influence on the structural properties of SP1 and SP2 imines
- the imine:TiO_2_ composites exhibited good air stability and reusability

The presented results support the conclusion that TiO_2_ changed the thermal, structural and electrochemical properties of the investigated imines and could be used in organic devices. However, more work is required concerning other organic compounds/polymers or other device architectures. This will be a challenge of our future work.

## Experimental

All chemicals and reagents were used as received from Sigma-Aldrich.

### Synthesis of TiO_2_

TiO_2_ powder was prepared by the sol–gel method using 4.5 mL TIPO dissolved in a solution of 20 mL ethanol and 3.5 mL distilled water (titania sol). During the stirring of the titania sol for about 4 h, TiO_2_ powder was introduced. The TiO_2_ powder was first filtered and dried at room temperature and then heated at 500 °C for one hour.

### Synthesis of imines

**Synthesis of (*E*,*E*)-(butane-1,4-diyl)bis(oxybutane-4,1-diyl) bis(4-{[(benzo[*d*][1,3]thiazol-2-yl)methylidene]amino}benzoate) (SP1).** Analogous to the procedure in [47] poly(butane-1,4-diol) bis(4-aminobenzoate) (1.0 mmol) and 1,3-benzothiazole-2-carbaldehyde (2.0 mmol) were added in a glass reactor fitted with a stirrer. The reaction mixture was purged with nitrogen for 30 min, and then the temperature was raised to 170 °C and kept on for 24 h under positive nitrogen pressure. The mixture was then cooled to room temperature, scraped and powdered. The crude product was washed three times with ethanol (3 × 500 mL) and then two times with acetone (2 × 300 mL) to remove unreacted compounds. Finally, the imine was dried at 60 °C under vacuum for 24 h to give the SP1 (70%).

^1^H NMR (400 MHz, CDCl_3_), δ [ppm]: 8.78 (s, 2H, –HC=N–), 8.16–8.06 (m, 6H), 7,96 (m, 2H), 7.84 (dd, 4H), 7.58–7.46 (m, 2H), 7.33 (m, 2H), 4.40–4.30 (m, 4H), 3.53–3.38 (m, 8H), 1.91–1.50 (m,12H).

**Synthesis of (*E*)-N-[(benzo[*d*][1,3]thiazol-2-yl)methylidene]-4-dodecylaniline (SP2).** As described elsewhere [51]: A mixture of 1,3-benzothiazole-2-carbaldehyde (1.0 mmol) and 4-dodecylaniline (1.0 mmol) in DMA solution in the presence of *p*-toluenesulfonic acid (PTS) (0.06 g) was refluxed with stirring for 10 h. The reaction was conducted in argon atmosphere and the condenser was fitted with a Dean–Stark trap. After cooling, the mixture was precipitated with 100 mL of ethanol. The crude product was washed three times with methanol (1500 mL) and then twice with acetone (800 mL) to remove unreacted monomers. Then the compound was dried at 60 °C for 12 h to give the SP2 (75%).

^1^H NMR (400 MHz, CDCl_3_), δ [ppm]: 8.82 (s, 1H, –**H**C=N–), 8.16 (dd, 1H), 7,95 (dd, 1H), 7.60 (m, 1H), 7.57–7.43 (m, 3H), 7.30 (d, 2H), 2.63 (t, 2H), 1.62 (m, 2H), 1.35–1.18 (m,18H), 0.87 (t, 3H).

### Preparation of imine:TiO_2_ mixture

To prepare the imine and TiO_2_ mixture, 3 mg of SP1 (or SP2) and a proper amount of TiO_2_ (anatase) were mixed in 1 mL of chloroform. The solution was dropped onto a microscope glass slide and heated at 60 °C for 3 h to evaporate the solvent. For POM measurements, the glass slide was covered with another glass, while for DSC studies, the mixture was scratched with a spatula and put into aluminum pans.

### Assembly and characterization of organic devices

Samples for photocurrent measurements were prepared on ITO patterned glass substrates (Osilla S211) to form ITO/TiO_2_/SP1 (or SP1:P3HT, SP1:TiO_2_, SP1:PC_71_BM or P3HT)/Au multilayer structures. First, the ITO-coated glass substrate was cleaned by ultrasonication in acetone and isopropanol for ≈20 min and oxygen plasma for 30 s. TiO_2_ (3Dnano P25, average particle size 21 ± 5 nm, mixed rutile (20%)/anatase (80%) phases, and a surface area of 50 m^2^/g) was mixed with ethanol for ≈6 h on a magnetic stirrer to form a homogeneous suspension. The prepared suspension was spun cast (750 rpm, 60 s) to form a uniform film and the TiO_2_ layer on ITO was annealed for 60 min at 650 °C. Then, various compound/mixtures in a chlorobenzene solution were spun cast on the top of the TiO_2_ layer. A gold electrode was deposited by thermal evaporation in vacuum (5 × 10^−6^ mbar). The current–voltage (*I*–*V*) characteristics were measured under illumination of a AM1.5 solar simulator (Oriel 150 W). The light power density was measured by a Newport Oriel P/N 91150V reference solar cell.

### Characterization techniques

The surface area and porosity analyzer by Micromeritics (ASAP2020) was used to obtain the physical parameters of titanium dioxide. The experiment was carried out at liquid nitrogen temperature, using nitrogen as an adsorbate. Prior to the measurements, the sample was precisely weighed (0.3248 g) and degassed at high vacuum for 8 h at 150 °C in order to remove the contamination. Adsorption–desorption isotherms were recorded at the pressure of 10^−5^ to 760 mmHg which was close to the saturation pressure of 1.0 *p*/*p*^0^. To calculate the surface area of TiO_2_ Brunauer–Emmett–Teller (BET) theory was applied while for porosity evaluation the Barrett–Joyner–Halenda (BJH) method was used. The wide angle X-ray diffraction patterns (WAXRD) were collected with a Bruker GADDS system (parallel beam of Cu Kα radiation formed by Goebel mirror monochromator and 0.5 mm point collimator, area detector VANTEC 2000) equipped with a modified Linkam heating stage. The samples were prepared in the form of a droplet on a heated surface. The textures of imines and their mixtures with TiO_2_ were registered by using a Nikon Eclipse LV100 POL polarizing microscope equipped with a Fine Instruments WTMS-14C heating stage. A Perkin Elmer DSC8000 calorimeter was used for calorimetric measurements. The temperature calibration procedure was done based on the onset of the melting point of water and indium. The sample was hermetically sealed in an aluminum pan of 30 μL. The temperature-dependent Fourier transform middle-infrared absorption measurements (FT-MIR) were performed using a Bruker VERTEX 70v vacuum spectrometer equipped with an Advanced Research System liquid helium DE-202A cryostat and water cooled helium compressor (ARS-2HW) working in a closed cycle manner. The set temperature was measured with an accuracy of ±0.1 K and stabilized for ≈3 min before the measurements were taken. The spectra were carried out in the spectral range of 4000–500 cm^−1^ with a resolution of 2 cm^−1^ and 32 scans per each spectrum. The SP1 and SP2 samples were mixed with KBr and then compressed into pellets before measurements. In the first step, the spectra were registered for every sample during heating and then in the second step during subsequent cooling. The SP1 sample was measured in the temperature range between −123 and 100 °C, and the SP2 sample between 20 and 90 °C. Additionally, room-temperature FT-MIR spectra of TiO_2_ (anatase form), SP1:TiO_2_ and SP2:TiO_2_ mixtures were obtained using the same spectrometer and spectral parameters. All spectra were recorded using OPUS 7.0 software and plotted with Origin 2017 Pro. The samples were characterized with ^1^H NMR, using deuterated chloroform (CDCl_3_) as a solvent with a Jeol ECZ-400 S spectrometer (1H - 400 MHz) with a delay time of 5 s. The measurements were carried out at room temperature on 10–15% (w/v) sample solutions. UV–vis spectra of SP1–SP2 compounds in a chloroform solution were recorded on a Jasco V670 spectrophotometer. AFM imaging of sample topography was measured at room temperature with the Agilent 5500 microscope working in non-contact mode. The setpoint and gains were adjusted to each measurement to obtain a clear image without noise. For each sample, topography images were collected at several randomly chosen areas. As described in [52], electrochemical measurements were carried out using an Eco Chemie Autolab PGSTAT128n potentiostat, glassy carbon electrode (diameter 2 mm), platinum coil and silver wire as working, auxiliary and reference electrodes, respectively. The potentials are referenced with respect to ferrocene (Fc), which was used as the internal standard. Cyclic voltammetry experiments were conducted in a standard one-compartment cell, in CH_2_Cl_2_ (Carlo Erba, HPLC grade), under argon. 0.2 M Bu_4_NPF_6_ (Aldrich, 99%) was used as the supporting electrolyte. The compound concentration was 1.0 × 10^−6^ mol/dm^3^. Deaeration of the solution was achieved by argon bubbling through the solution for about 10 min before measurement. All electrochemical experiments were carried out under ambient conditions.
